# Quality of systematic reviews and meta‐analyses in dermatology

**DOI:** 10.1002/cesm.12056

**Published:** 2024-05-02

**Authors:** Annapoorani Muthiah, Loch Kith Lee, John Koh, Ashly Liu, Aidan Tan

**Affiliations:** ^1^ School of Clinical Medicine, Faculty of Medicine and Health University of New South Wales Sydney New South Wales Australia

**Keywords:** dermatology, meta‐analysis, methodological quality, reporting quality, risk of bias, systematic review

## Abstract

**Introduction:**

Although the number of published systematic reviews and meta‐analyses in dermatology has increased over the past decade, their quality is unknown.

**Objective:**

The objective of this study was to determine the change in risk of bias, methodological quality and reporting quality of systematic reviews and meta‐analyses in dermatology between 2010 and 2019.

**Methods:**

We conducted a comparative study of systematic reviews and meta‐analyses published in the 10 highest‐ranked dermatology journals in 2010 and 2019. Studies were identified through electronic searches of MEDLINE, Embase, and eight other bibliographic databases. Risk of bias and methodological quality were assessed in duplicate with the risk of bias in systematic reviews (ROBIS) and A MeaSurement Tool to Assess systematic Reviews‐2 (AMSTAR‐2) tools, respectively, with the latter only applied to studies of interventions. Reporting quality was assessed with the Preferred Reporting Items of systematic reviews and Meta‐Analyses (PRISMA) 2009 and PRISMA for abstracts (PRISMA‐A) 2013 statements.

**Results:**

We included 27 systematic reviews and meta‐analyses published in 2010 and 127 published in 2019. There was no evidence of a difference in the proportion of systematic reviews and meta‐analyses at high/unclear risk of bias with ROBIS (Fisher's exact test = 1.00) or critically low methodological quality using AMSTAR‐2 (Fisher's exact test = 0.456), between 2010 and 2019. There was evidence of a difference in proportion of PRISMA (*t*(26) = 2.7, *p* = 0.01), and very strong evidence of a difference in proportion of PRISMA‐A (*t*(26) = 4.2, *p* < 0.001) checklist items adequately reported between 2010 and 2019. The difference in mean proportion of PRISMA checklist items adequately reported was 3.6 items more (95% confidence interval [CI]: 1.8–5.4 items more) in 2019 (mean = 10.7 items, SD = 2.4 items) than in 2010 (mean = 7.1 items, SD = 2.9 items), and of PRISMA‐A checklist items adequately reported was 1.1 items more (95% CI: 0.2–2.0 items more) in 2019 (mean = 5.6 items, SD = 1.5 items) than in 2010 (mean = 4.4 items, SD = 1.7 items).

**Conclusions:**

No improvement was observed in the overall methodological quality of included systematic reviews and meta‐analyses; however, there was strong evidence of improvement in the overall reporting quality.

## Plain Language Summary

Systematic reviews are a method of secondary research that combines findings from multiple primary studies to produce more reliable conclusions. These conclusions are used in various contexts, including to guide patient care and inform future research topics. The number of published systematic reviews in the field of dermatology has substantially increased in the last few years. However, it is unknown how well‐performed these systematic reviews are. Our aim was to find out whether the quality of these systematic reviews has changed between 2010 and 2019. To do this, we appraised the systematic reviews published in the 10 highest‐ranking dermatology journals in 2010 and 2019, using tools designed to assess the quality of systematic reviews — specifically, the “'Risk Of Bias In Systematic reviews' (ROBIS) and 'A MeaSurement Tool to Assess systematic Reviews‐2' (AMSTAR‐2) tools. All included systematic reviews were assessed by two different authors independently. We also assessed if all essential components of the systematic reviews were reported transparently and completely to allow the reader to review accurately and interpret the systematic review and its findings. For this, we used the 'Preferred Reporting Items of Systematic reviews and Meta‐Analyses' (PRISMA) and 'PRISMA for Abstracts' (PRISMA‐A) statements.

## 1 INTRODUCTION

### 1.1 Background

Systematic reviews answer specific research questions by synthesizing existing primary evidence in a manner that minimizes bias [1, 2, 3, 4]. This sometimes involves meta‐analysis, a statistical method to combine results of discrete but similar studies [4, 5]. Systematic reviews and meta‐analyses heavily inform clinical guidelines, physician practice, and policy decisions [2, 6, 7].

**Figure 1:**
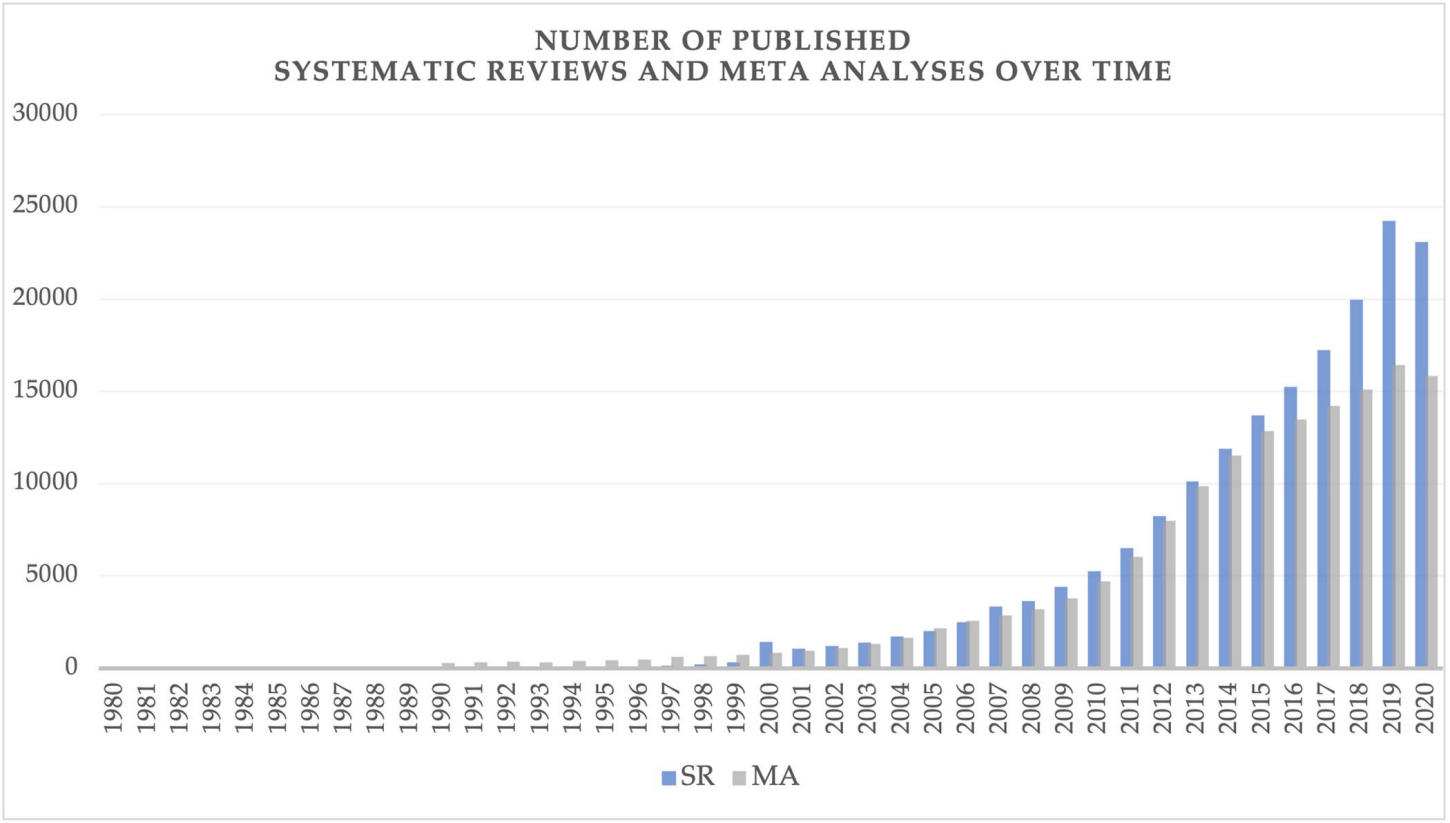
Number of PubMed‐indexed articles published each year between 1986 and 2014 with the tag “systematic review” or “meta‐analysis” for the type of publication [8].

In recent decades, there has been an increasing number of published systematic reviews and meta‐analyses (Figure 1) [2, 8]. Between 1991 and 2014, the total number of PubMed items increased by 153%, but the number of systematic reviews and meta‐analyses increased by 2.728% and 2.635%, respectively [8]. Although a proportion of recently published systematic reviews meet the demands of expanding primary literature [9], there is also increasing redundancy among systematic reviews, which address the same topics without new insight [8, 10], or lead to contradictory results [11, 12]. This has been demonstrated in multiple disciplines, including surgery [11], orthopedics [12], hepatology [13], and cardiology [10]. Some studies have attributed the redundancy and conflict between systematic reviews to differences in the quality and conduct [11, 12, 14].

There are serious concerns about the risk of bias [12, 15, 16], methodological quality [13, 17], and reporting quality [17, 18] of systematic reviews, and the overuse and reliance on systematic reviews without critically reflecting on their conduct. Risk of bias, as assessed by the risk of bias in systematic reviews (ROBIS) tool [19], was found to be unclear or high in most systematic reviews evaluated in several studies, with particular problems with study selection [12, 15, 16]. Methodological quality of systematic reviews in hepatology, as assessed by the A MeaSurement Tool to Assess systematic Reviews (AMSTAR) tool [20], was found to be particularly poor in the risk of bias assessment of primary studies, consideration of risk of bias of primary studies in drawing systematic review conclusions, and assessment of publication bias [13]. Studies of reporting quality, as assessed by the Preferred Reporting Items of systematic reviews and Meta‐Analyses (PRISMA) statement [3], have found that many items are reported by less than two‐thirds of systematic reviews [18]. Additionally, despite the stated use of reporting guidelines, author adherence to reporting guidelines is low to moderate [21, 22].

### 1.2 Objectives

Our primary aim was to assess the risk of bias and methodological quality of systematic reviews and meta‐analyses published in the 10 highest‐ranked dermatology journals in 2010 and 2019 to assess for change through a comparative study. Our secondary aim was to assess the reporting quality of included studies and similarly determine the change between the 2 years.

## 2 METHODS

### 2.1 Dermatology journals

Peer‐reviewed journals were eligible for inclusion if (a) their primary scope was dermatology, (b) they published at least one systematic review or meta‐analysis in either 2010 or 2019, and (c) they were established before 2010. The 10 highest‐ranked eligible journals by the SCImago Journal Rank were included [23]. This was used to consider the level of influence systematic reviews published in these journals would have, rather than as a measure of the quality of evidence published in these journals.

### 2.2 Systematic reviews and meta‐analyses

All articles published in included journals were identified by searching bibliographic databases and journal websites. The bibliographic databases searched were MEDLINE via PubMed, Embase, Cochrane Database of systematic reviews, ACP Journal Club, the Database of Abstracts of Reviews of Effectiveness, Cochrane Clinical Answers, Cochrane Central Register of Controlled Trials, Cochrane Methodology Register, Health Technology Assessments, and the NHS Economic Evaluation Database. All articles were screened by title and abstract to determine if the study was a systematic review or meta‐analysis. Systematic reviews and meta‐analyses were eligible for inclusion if they were published in (a) an included journal, (b) either 2010 or 2019, and (c) full text. Systematic reviews and meta‐analyses were excluded if (a) they were published as conference abstracts, letters to editors, or commentaries, (b) the authors explicitly differentiated the methodology of their study from that of a systematic review or meta‐analysis, or (c) the meta‐analysis was not the primary analysis reported in the article, but rather the analysis of more than one primary study conducted by the authors and reported in the same article.

### 2.3 Data source

Study characteristics extracted related to the methods, conduct, results, conclusions, and references. Data on the risk of bias tools used to assess individual studies and the databases searched was also extracted (Supporting Information S1: Table S1). Author characteristics extracted included the country of first author and number of listed authors. Journal characteristics extracted included endorsement of PRISMA statement and requirements for PROSPERO registration (Supporting Information S1: Table S2).

### 2.4 Data collection

Two investigators independently assessed the risk of bias of each systematic review with the ROBIS tool, and the methodological quality of each systematic review of interventions with the AMSTAR‐2 tool. Any discrepancies were resolved by discussion with a third investigator. All investigators applied the tools in strict accordance with guidelines. One investigator assessed the reporting quality of systematic reviews and their abstracts using the PRISMA [3] and PRISMA for abstracts (PRISMA‐A) [24] checklists, respectively.

To additionally validate the ROBIS and AMSTAR‐2 assessments, we compared our assessments with those of other authors. This involved searching the Web of Science database for all articles, which cited systematic reviews included in our study, screening the titles for systematic reviews or meta‐analyses, and comparing other authors' assessments with our own.

### 2.5 Statistical analysis

Statistical analysis was performed with jamovi 1.6.23 [25]. Categorical variables were summarized with frequencies, and continuous variables were summarized with mean and standard deviation, or median and interquartile range, where applicable. Reporting within individual systematic reviews and meta‐analyses between 2010 and 2019 were compared with independent samples *t*‐test and difference in means with 95% confidence intervals (CIs). Reporting of individual PRISMA‐A and PRISMA checklist items between 2010 and 2019 were compared with *χ* ^2^ test for independent proportions, or Fisher's exact test if any of the expected frequencies were less than 5, and difference in proportions with 95% CIs. Domain‐specific and overall risk of bias within individual systematic reviews and meta‐analyses between 2010 and 2019 were compared using *χ* ^2^ test for independent samples, or Fisher's exact test if any of the expected frequencies were less than 5, and difference in proportions with 95% CIs.

## 3 RESULTS

### 3.1 Article characteristics

We included 27 systematic reviews and meta‐analyses published in 2010 and 127 published in 2019. The most common dermatological subspecialty, country of first author, and primary funding source were similar for systematic reviews published in both 2010 and 2019, as well as the median number of authors, databases searched, sample size, and references (Supporting Information S1: Table S1). The most studied dermatological disease for both years was psoriasis. No systematic review from 2010 was registered and only 29.9% (*n* = 38/127) were registered in 2019 (Supporting Information S1: Table S1). In the title, 56% of included 2010 studies and 43% of included 2019 studies identified as a systematic review, and 7.4% of included 2010 studies and 42.5% of included 2019 studies identified as both a systematic review and meta‐analysis. Additionally, 29.6% of the 2010 systematic reviews and 44.1% of the 2019 systematic reviews were of interventions.

### 3.2 Journal characteristics

The median impact factor of included journals was 3.81 (interquartile range [IQR]: 1.24) in 2010 and 5.33 (IQR: 3.66) in 2019. The median number of publications per journal was 315 (IQR: 270) in 2010 and 802 (IQR: 1125) in 2019. The peer‐review process was single‐blinded for seven journals, double‐blinded for one journal, and unspecified, on their website, and in response to email inquiries, for two journals. Two journals required PRISMA, five recommended PRISMA, and three had no mention of PRISMA. Two journals explicitly recommended PROSPERO registration, five implicitly recommended registration, and three did not mention any recommendations for PROSPERO.

### 3.3 Reporting quality

#### 3.3.1 Overall

There was strong evidence of a difference in the proportion of PRISMA checklist items adequately reported between 2010 and 2019 (*t*(26) = 2.7, *p* = 0.01). The difference in mean proportion of PRISMA checklist items adequately reported was 3.6 items more (95% CI: 1.8–5.4 items more) in 2019 (mean = 10.7 items, SD = 2.4 items) than in 2010 (mean = 7.1 items, SD = 2.9 items).

The items with a strong difference in reporting between 2010 and 2019 include that of study selection in the methods (item 9) (*χ* ^2^(1) = 21.9, *p* < 0.001) with 36.2% more systematic reviews adequately reporting this item (95% CI: 17.5–54.8) in 2019 (87%) than in 2010 (48.1%), and protocol and registration in the methods (item 5) (*χ* ^2^(1) = 8.45, *p* = 0.004) (Table 1). There was very strong evidence of a difference in the proportion of systematic reviews and meta‐analyses adequately reporting study selection in the results (item 17) between 2010 and 2019 (Fisher's exact test, *p* < 0.001). The difference in the proportion of systematic reviews and meta‐analyses adequately reporting study selection in the results was 55.1% more (95% CI: −73.9 to −36.3) in 2019 (92.1%) than in 2010 (37%). There was also strong evidence of a difference in the proportion of systematic reviews and meta‐analyses adequately reporting search strategy in the methods (*χ* ^2^(1) = 6.59, *p* = 0.01) and risk of bias within studies in the results (*χ* ^2^(1) = 7.16, *p* = 0.007) between 2010 and 2019. The difference in the proportion of systematic reviews and meta‐analyses adequately reporting risk of bias within studies in the results was 16.6% more (95% CI: 5.58–27.7) in 2019 (46%) than in 2010 (18.5%).

**Table 1:** Reporting of PRISMA checklist items of systematic reviews and meta‐analyses in peer‐reviewed dermatology journals in 2010 (*N* = 27) and 2019 (*N* = 127).

PRISMA checklist item	2010, *n* (%)	2019, *n* (%)	*χ* ^2^ test for independent proportion/Fisher's exact test^a^	Difference in proportions % (95% CI)
*Title*				
Title				
Yes	21 (77.7)	115 (91.0)	Fisher's exact test, *p* = 0.092	−17.9 (−40.5 to 4.72)
No	6 (22.2)	12 (9.0)		
*Abstract*				
Structured summary				
Yes	20 (74.0)	102 (80.0)	*χ* ^2^(1) = 0.52, *p* = 0.468	5.4 (−10.3 to 21.2)
No	7 (25.9)	25 (20.0)		
*Introduction*				
Rationale				
Yes	23 (85.1)	121 (95.0)	Fisher's exact test, *p* = 0.075	24 (−6.92 to 55.0)
No	4 (14.8)	6 (5.0)		
Objectives				
Yes	24 (88.8)	123 (97.0)	Fisher's exact test, *p* = 0.104	26.5 (−10.6 to 63.7)
No	3 (11.1)	4 (3.0)		
*Methods*				
Protocol and registration				
Yes	1 (3.7)	39 (31.0)	*χ* ^2^(1) = 8.45, *p* = 0.004	20.3 (11.2–29.4)
No	26 (96.2)	88 (69.0)		
Eligibility criteria				
Yes	5 (18.5)	33 (26.0)	*χ* ^2^(1) = 0.668, *p* = 0.414	5.81 (−7.09 to 18.7)
No	22 (81.4)	94 (74.0)		
Information sources				
Yes	18 (66.6)	109 (86.0)	Fisher's exact test, *p* = 0.026	19.2 (0.37–37.9)
No	9 (33.3)	18 (14.0)		
Search strategy				
Yes	10 (37.0)	81 (64.0)	*χ* ^2^(1) = 6.59, *p* = 0.01	16 (3.29–28.7)
No	17 (62.9)	46 (36.0)		
Study selection				
Yes	13 (48.1)	111 (87.0)	*χ* ^2^(1) = 21.9, *p* < 0.001	36.2 (17.5– 54.8)
No	14 (51.8)	16 (13.0)		
Data collection process				
Yes	19 (70.3)	87 (69.0)	*χ* ^2^(1) = 0.03, *p* = 0.849	−1.26 (−14 to 11.6)
No	8 (29.6)	40 (31.0)		
Data items				
Yes	18 (66.6)	87 (69.0)	*χ* ^2^(1) = 0.03, *p* = 0.852	1.22 (−11.8 to 14.2)
No	9 (33.3)	40 (31.0)		
Risk of bias in individual studies				
Yes	2 (7.4)	16 (13.0)	Fisher's exact test, *p* = 0.741	7.27 (−8.64 to 23.2)
No	25 (92.5)	111 (87.0)		
Summary measures				
Yes	14 (51.8)	70 (55.1)	Fisher's exact test, *p* = 0.583	4.62 (−31.3 to 40.6)
No	1 (3.7)	4 (3.1)		
Not applicable	12 (44.4)	53 (41.7)		
Synthesis of results				
Yes	13 (48.1)	61 (48.0)	Fisher's exact test, p = 1.00	−6.44 (−26.9 to 14.0)
No	2 (7.4)	9 (7.0)		
Not applicable	12 (44.4)	57 (44.8)		
Risk of bias across studies				
Yes	6 (22.2)	35 (28.0)	*χ* ^2^(1) = 0.325, *p* = 0.569	3.95 (−9.03 to 16.9)
No	21 (77.7)	92 (72.0)		
Additional analyses				
Yes	2 (7.4)	18 (14.0)	Fisher's exact test, *p* = 0.53	8.6 (−6.05 to 23.4)
No	25 (92.5)	109 (86.0)		
*Results*				
Study selection				
Yes	10 (37.0)	117 (92.1)	Fisher's exact test, *p* = 0.001	−55.1 (−73.9 to −36.3)
No	17 (62.9)	10 (7.8)		
Study characteristics				
Yes	17 (62.9)	93 (73.0)	*χ* ^2^(1) = 1.15, *p* = 0.284	7.27 (−6.83 to 21.4)
No	10 (37.0)	34 (27.0)		
Risk of bias within studies				
Yes	5 (18.5)	59 (46.0)	*χ* ^2^(1) = 7.16, *p* = 0.007	16.6 (5.58 to 27.7)
No	22 (81.4)	68 (54.0)		
Results of individual studies				
Yes	9 (33.3)	63 (50.0)	Fisher's exact test, *p* = 0.219	15.5 (9.43 to 40.5)
No	6 (22.2)	10 (7.8)		
Not applicable	12 (44.4)	54 (42.5)		
Synthesis of results				
Yes	13 (48.1)	61 (48.0)	*χ* ^2^(1) = 0.71, *p* = 0.398	10 (−13 to 32)
No	2 (7.4)	9 (7.0)		
Not applicable	12 (44.4)	57 (44.8)		
Risk of bias across studies				
Yes	5 (18.5)	30 (24.0)	χ^2^(1) = 0.33, *p* = 0.566	4.2 (−9.33 to 17.7)
No	22 (81.4)	97 (76.0)		
Additional analysis				
Yes	5 (18.5)	41 (32.0)	*χ* ^2^(1) = 2.01, *p* = 0.156	9.5 (−2.27 to 21.3)
No	22 (81.4)	86 (68.0)		
*Discussion*				
Summary of evidence				
Yes	14 (67.0)	75 (59.0)	*χ* ^2^(1) = 0.474, *p* = 0.491	4.27 (−8.05 to 16.6)
No	13 (62.0)	52 (41.0)		
Limitations				
Yes	8 (38.0)	56 (44.0)	*χ* ^2^(1) = 1.92, *p* = 0.166	8.61 (−3.08 to 20.3)
No	19 (70.3)	71 (56.0)		
Conclusions				
Yes	18 (66.6)	87 (69.0)	*χ* ^2^(1) = 0.034, *p* = 0.852	1.22 (−11.8 to 14.2)
No	9 (33.3)	40 (31.0)		
*Funding*				
Funding				
Yes	23 (85.1)	100 (79.0)	*χ* ^2^(1) = 0.575, *p* = 0.448	−5.8 (−19.5 to 7.87)
No	4 (14.8)	27 (21.0)		

*Note*: *n* = count; % = percentage; *χ* ^2^ = chi‐squared.

Abbreviations: CI, confidence intervals; PRISMA, Preferred Reporting Items of systematic reviews and Meta‐Analyses.

^a^ Fisher's exact test was used if any of the expected frequencies were less than 5.

#### 3.3.2 Abstract

As assessed by the PRISMA checklist, there was no evidence of a difference in the proportion of systematic reviews and meta‐analyses adequately reporting a structured summary in the abstract between 2010 and 2019 (Table 1). However, there was strong evidence of a difference in the proportion of PRISMA‐A checklist items adequately reported between 2010 and 2019 (*t*(26) = 4.2, *p* < 0.001), with 1.1 more PRISMA‐A checklist items (95% CI: 0.2–2.0 items more) being adequately reported in 2019 (mean = 5.6 items, SD = 1.5 items) than 2010 (mean = 4.4 items, SD = 1.7 items).

There was strong evidence of a difference in the proportion of systematic reviews and meta‐analyses adequately reporting the objectives in the abstract between 2010 and 2019 (Fisher's exact test, *p* = 0.003), as well as in the reporting of information sources in the abstract between 2010 and 2019 (*χ* ^2^(1) = 3.86, *p* = 0.05) (Table 2). The difference in the proportion of systematic reviews and meta‐analyses adequately reporting the objectives in the abstract was 64.6% (95% CI: 29–100) more in 2019 (99%) than in 2010 (85.1%). The difference in the proportion of systematic reviews and meta‐analyses adequately reporting information sources in the abstract was 12.3% (95% CI: 1–23.7) more in 2019 (43%) than in 2010 (22.2%).

**Table 2:** Reporting of PRISMA‐A checklist items of systematic reviews and meta‐analyses in peer‐reviewed dermatology journals in 2010 (*N* = 27) and 2019 (*N* = 127).

PRISMA‐A checklist item	2010, *n* (%)	2019, *n* (%)	*χ* ^2^ test for independent proportion/Fisher's exact test^[Link]^, ^a^	Difference in proportions % (95% CI)
*Title*				
Title				
Yes	21 (77.7)	115 (91.0)	Fisher's exact test, *p* = 0.092	−17.9 (−40.5 to 4.72)
No	6 (22.2)	12 (9.0)		
Background				
Objectives				
Yes	23 (85.1)	126 (99.0)	Fisher's exact test, *p* = 0.003	64.6 (29.0 to 100.0)
No	4 (14.8)	1 (1.0)		
*Methods*				
Eligibility criteria				
Yes	6 (22.2)	21 (17.0)	Fisher's exact test, *p* = 0.577	−5.69 (−22.6 to 11.3)
No	21 (77.7)	106 (83.0)		
Information sources				
Yes	6 (22.2)	54 (43.0)	*χ* ^2^(1) = 3.86, *p* = 0.05	12.3 (1.0 to 23.7)
No	21 (77.7)	73 (57.0)		
Risk of bias				
Yes	3 (11.1)	21 (17.0)	Fisher's exact test, *p* = 0.574	5.96 (−8.86 to 20.8)
No	24 (88.8)	106 (83.0)		
*Results*				
Included studies				
Yes	8 (29.6)	30 (24.0)	*χ* ^2^(1) = 0.432, *p* = 0.511	−4.67 (−19.3 to 9.93)
No	19 (70.3)	97 (76.0)		
Synthesis of results				
Yes	22 (81.4)	108 (85.0)	Fisher's exact test, *p* = 0.77	3.91 (−13.6 to 21.4)
No	5 (18.5)	19 (15.0)		
Description of the effect				
Yes	18 (66.6)	88 (69.0)	*χ* ^2^(1) = 0.071, *p* = 0.789	1.77 (−11.4 to 14.9)
No	9 (33.3)	39 (31.0)		
*Discussion*				
Strengths and limitations of evidence				
Yes	9 (33.3)	62 (49.0)	*χ* ^2^(1) = 2.15, *p* = 0.143	9.01 (−2.76 to 20.8)
No	18 (66.6)	65 (51.0)		
Interpretation				
Yes	26 (96.2)	120 (94.0)	Fisher's exact test, *p* = 1.00	−5.31 (−29.1 to 18.4)
No	1 (3.7)	7 (6.0)		
*Other*				
Funding				
Yes	0	0	‐	‐
No	27 (100.0)	127 (100.0)		
Registration				
Yes	0	5 (4.0)	Fisher's exact test, *p* = 0.587	18.1 (11.9 to 24.3)
No	27 (100.0)	122 (96.0)		

*Note*: *n* = count; % = percentage; *χ* ^2^ = chi‐squared.

Abbreviations: CI, confident intervals; PRISMA‐A, Preferred Reporting Items of systematic reviews and Meta‐Analyses for abstracts.

^a^ Fisher's exact test was used if any of the expected frequencies were less than 5.

### 3.4 ROBIS

There was evidence of a difference in the proportion of systematic reviews and meta‐analyses with high/unclear eligibility bias, between 2010 and 2019 (*χ* ^2^(1) = 8.65, *p* = 0.003). The difference in the proportion of systematic reviews and meta‐analyses with high eligibility bias was 31% more (95% CI: 11.8–50.2) in 2010 (70.3%) than in 2019 (39.3%). There was little to no evidence, however, of a difference in the proportion of systematic reviews and meta‐analyses with high/unclear overall risk of bias, between 2010 and 2019 (Fisher's exact test = 1) (Table 3).

**Table 3:** Domain‐specific risk of bias (ROBIS tool) of individual systematic reviews and meta‐analyses in 2010 (*N* = 27) and 2019 (N = 127).

Domain‐specific risk of bias	2010, *n* (%)	2019, *n* (%)	*χ* ^2^ test for independent proportion^a^/Fisher's exact test^b^	Difference in proportions % (95% CI)
Eligibility bias				
Low	8 (29.6)	77 (60.6)	*χ* ^2^(1) = 8.65, *p* = 0.003	31.0 (11.8–50.2)
Unclear	6 (22.2)	2 (1.5)		
High	13 (48.1)	48 (37.7)		
Identification bias				
Low	6 (22.2)	41 (32.2)	*χ* ^2^(1) = 1.06, *p* = 0.303	10.1 (−7.6 to 27.7)
Unclear	4 (14.8)	0		
High	17 (62.9)	86 (67.7)		
Collection bias				
Low	6 (22.2)	27 (21.2)	*χ* ^2^(1) = 0.0122, *p* = 0.912	−0.96 (−18.2 to 16.3)
Unclear	4 (14.8)	2 (1.5)		
High	17(62.9)	98 (77.1)		
Synthesis bias				
Low	7 (25.9)	15 (11.8)	Fisher's exact test, *p* = 0.07	−14.1 (−31.6 to 3.34)
Unclear	4 (14.8)	2 (1.5)		
High	16 (59.2)	110 (86.6)		
Overall bias				
Low	5 (18.5)	22 (17.3)	Fisher's exact test, *p* = 1	−1.20 (−17.3 to 14.9)
Unclear	2 (7.4)	1 (0.7)		
High	20 (74.0)	104 (81.8)		

*Note*: *n* = count; % = percentage; *χ* ^2^ = chi‐squared.

Abbreviations: CI, confident intervals; ROBIS, risk of bias in systematic reviews.

^a^ *χ* ^2^ independent test of proportions was performed with high and unclear risk of bias merged into one outcome variable.

^b^ Fisher's exact test was used if any of the expected frequencies were less than 5.

### 3.5 AMSTAR‐2

There was little to no evidence of a difference in the proportion of systematic reviews and meta‐analyses with critically low methodological quality, between 2010 and 2019 (Fisher's exact test = 0.456) (Table 4). The difference in the proportion of systematic reviews and meta‐analyses with critically low methodological quality was −12.9% more (95% CI: −56.2 to 0.3) in 2019 (94.4%) than in 2010 (87.5%).

**Table 4:** Item‐specific risk of bias (AMSTAR‐2 tool) of individual systematic reviews and meta‐analyses in 2010 (*N* = 8) and 2019 (*N* = 54).

AMSTAR item	2010, *n* (%)	2019, *n* (%)	*χ* ^2^ test for independent proportion/Fisher's exact test^[Link]^, ^a^	Difference in proportions % (95% CI)
Overall methodological quality				
Critically low	7 (87.5)	51 (94.4)	Fisher's exact test, *p* = 0.456	−12.9 (−56.2 to 30.3)
Low	1 (14.2)	2 (3.7)		
Moderate	0	1 (1.8)		
High	0	0		
Q1. Did the research questions and inclusion criteria for the review include the components of PICO?				
Yes	7 (87.5)	34 (62.9)	Fisher's exact test, *p* = 0.171	
No	1 (12.5)	20 (37.1)		
Q2. Did the report of the review contain an explicit statement that the review methods were from the protocol?				
Yes	1 (14.2)	10 (18.5)	Fisher's exact test, *p* = 0.367	
Partial yes	0	9 (16.6)		
No	7 (87.5)	35 (64.8)		
Q3. Did the review authors explain their selection of the study designs for inclusion in the review?				
Yes	3 (37.5)	10 (18.5)	Fisher's exact test, *p* = 0.218	
No	5 (62.5)	44 (81.4)		
Q4. Did the review authors use a comprehensive literature search?				
Yes	3 (37.5)	0	Fisher's exact test, *p* < 0.001	
Partial yes	3 (37.5)	18 (33.3)		
No	2 (25.0)	36 (66.6)		
Q5. Did the review authors perform study selection in duplicate?				
Yes	7 (87.5)	32 (59.2)	Fisher's exact test, *p* = 0.123	
No	1 (14.2)	22 (40.7)		
Q6. Did the review authors perform data extraction in duplicate?				
Yes	6 (85.7)	31 (57.4)	Fisher's exact test, *p* = 0.344	
No	2 (25.0)	23 (42.5)		
Q7. Did the review authors provide a list of excluded studies and justify the exclusions?				
Yes	1 (14.2)	6 (11.1)	Fisher's exact test, *p* = 0.923	
Partial yes	0	1(1.8)		
No	7 (87.5)	47 (87.0)		
Q8. Did the review authors describe the included studies in adequate detail?				
Yes	3 (37.5)	9 (16.6)	Fisher's exact test, *p* = 0.218	
Partial yes	3 (37.5)	15 (27.7)		
No	2 (25.0)	30 (55.5)		
Q9. RCT: Did the review authors use a satisfactory technique for assessing the RoB in individual studies that were included in the review?				
Yes	4 (0.5)	25 (46.2)	Fisher's exact test, *p* = 0.068	
Partial yes	1 (14.2)	0		
No	2 (25.0)	20 (37.0)		
Includes only NRSIs	1 (14.2)	9 (16.6)		
Q9. NRSI: Did the review authors use a satisfactory technique for assessing the RoB in individual studies that were included in the review?				
Yes	1 (14.2)	3 (5.5)	Fisher's exact test, *p* = 0.189	
Partial yes	0	6 (11.1)		
No	2 (25.0)	29 (53.7)		
Includes only RCTs	5 (62.5)	16 (29.6)		
Q10. Did the review authors report on the sources of funding for the studies included in the review?				
Yes	3 (37.5)	2 (3.7)	Fisher's exact test, *p* = 0.001	
No	5 (62.5)	52 (96.2)		
MAQ11. RCT: If meta‐analysis was performed did the review authors use appropriate methods for statistical combination of results?				
Yes	5 (62.5)	10 (18.5)	Fisher's exact test, *p* = 0.019	
No	0	12 (22.2)		
No MA conducted	3 (37.5)	32 (59.2)		
MAQ11: NRSI: If meta‐analysis was performed did the review authors use appropriate methods for statistical combination of results?				
Yes	0	0	Fisher's exact test, *p* = 0.102	
No	0	14 (25.9)		
No MA conducted	8 (100.0)	40 (74.0)		
MAQ12. If meta‐analysis was performed, did the review authors assess the potential impact of RoB in individual studies on the results of the meta‐analysis or other evidence synthesis?				
Yes	3 (37.5)	7 (12.9)	Fisher's exact test, *p* = 0.201	
No	2 (25.0)	23 (42.5)		
No MA conducted	3 (37.5)	24 (44.4)		
Q13. Did the review authors account for RoB in individual studies when interpreting/discussing the results of the review?				
Yes	6 (75.0)	18 (33.3)	Fisher's exact test, *p* = 0.024	
No	2 (25.0)	36 (66.6)		
Q14. Did the review authors provide a satisfactory explanation for, and discussion of, any heterogeneity observed in the results of the review?				
Yes	6 (75.0)	23 (42.5)	Fisher's exact test, *p* = 0.086	
No	2 (25.0)	31 (57.4)		
MAQ15. If they performed quantitative synthesis did the review authors carry out an adequate investigation of publication bias (small study bias) and discuss its likely impact on the results of the review?				
Yes	3 (37.5)	8 (14.8)	Fisher's exact test, *p* = 0.280	
No	2 (25.0)	22 (40.7)		
No MA conducted	3 (37.5)	24 (44.4)		
Q16. Did the review authors report any potential sources of conflict of interest, including any funding they received for conducting the review?				
Yes	8 (100.0)	54 (100.0)	Fisher's exact test, *p* < 0.001	
No	0	0		

*Note*: *n* = count; % = percentage; *χ* ^2^ = chi‐squared.

Abbreviations: AMSTAR‐2, A MeaSurement Tool to Assess systematic Reviews‐2; CI, confidence intervals; MA, meta‐analysis; NRSI, non‐randomised studies of interventions; PICO, patient/population, intervention, comparison, outcome; RCT, randomized controlled trial; ROB, risk of bias.

^a^ Fisher's exact test was used if any of the expected frequencies were less than 5.

### 3.6 Validation check

We identified seven studies that included a total of 12 systematic reviews we assessed in our study. We were unable to compare the assessment of methodological quality for eight of these: six because the authors used AMSTAR‐2 for noninterventional systematic reviews, and two because the authors did not provide their assessment results. Of the remaining four, there was an agreement rate of 87.5% between our and other authors' assessment of individual items. This is higher than the inter‐rater reliability scores reported in the literature [26, 27].

### 3.7 Agreement between raters

The overall interrater agreement was 85% for AMSTAR‐2 and 83% for ROBIS, which is higher than other studies utilizing these tools [1, 27, 28].

## 4 DISCUSSION

### 4.1 Key results

There is little to no significant difference in the proportion of systematic reviews with high risk of bias or critically low methodological quality between 2010 and 2019. There is strong evidence of improvement in the reporting quality of systematic reviews from 2010 to 2019.

### 4.2 Risk of bias and methodological quality

More than four in five systematic reviews and meta‐analyses published in the 10 highest‐ranked dermatology journals in both 2010 and 2019 were at high risk of bias (Table 3). Our results build on Smires et al.'s study of systematic reviews in dermatology published in 2017 and demonstrate that poor methodological quality in systematic reviews and meta‐analyses in dermatology have persisted over time [29].

Although there was an improvement in unnecessary restrictions on study eligibility criteria, all other risk of bias domains remained unchanged. Most systematic reviews and meta‐analyses in 2019 failed to search gray literature and other unpublished works, potentially contributing to publication bias [30]. This may be related to challenges in identifying gray literature owing to limited centralized databases, and search strategies needing to be modified for each database [31]. The key concern for systematic reviews and meta‐analyses in 2010 was the lack of formal risk of bias assessment. By comparison, the key concern for systematic reviews and meta‐analyses in 2019 was the paucity of available study characteristics, although it is unclear how much of this is attributable to limits on word count or supplementary material [18]. Most systematic reviews and meta‐analyses in both 2010 and 2019 inadequately considered biases in primary studies and failed to deliberate study limitations when discussing review findings, despite this being a core component of evidence synthesis.

Most systematic reviews and meta‐analyses in 2019 (*n* = 51/54, 94.4%) and 2010 (*n* = 7/8, 87.5%) had critically low methodological quality (Table 4). In meta‐research studies that included Cochrane reviews, a lower proportion of critically low methodological quality systematic reviews has been reported [32, 33]. Of systematic reviews and meta‐analyses in 2019, none fully satisfied and only a third partially satisfied the criteria for use of a comprehensive search strategy. This has only been reported by one other study [34], with still other studies finding the use of a comprehensive search strategy to be more frequently satisfactory [32, 35].

In comparison to our study, Gómez‐García et al. found that 86% of systematic reviews on psoriasis interventions were at high risk of bias as assessed by ROBIS and 24.5% had low methodological quality as assessed by AMSTAR [7], although it might be possible to achieve higher quality scores with AMSTAR than AMSTAR‐2 [36].

### 4.3 Reporting quality of systematic reviews

Systematic reviews and meta‐analyses in 2019 had better overall reporting, with a mean of 3.6 more PRISMA items reported in 2019 compared to 2010. Our results also correspond with Croitoru et al.'s study of systematic reviews in dermatology between 2013 and 2017, which found improved adherence to PRISMA over time [37]. However, this study only assessed for adherence to PRISMA items relevant to methodology [37]. This improvement may be due to journal endorsement of PRISMA guidelines, which were introduced in 2009 [21], with seven of the 10 journals studied requiring or recommending adherence to PRISMA in 2019. Journal endorsement of reporting guidelines may have broader implications, with a previous study even finding that endorsement of PRISMA led to higher methodological quality as assessed by AMSTAR [38]. These improvements may depend on whether endorsing journals require or recommend adherence [39]; however, the inconsistency in implementation across journals makes the relationship difficult to establish [40].

One area with improvement, protocol registration, remains insufficiently reported, with only 31% of included studies in 2019 reporting registration. This is an improvement to the previously reported 15% registration rate for systematic reviews in dermatology [29]. Remarkably, there has been no improvement in the reporting of funding of studies, with 79% of systematic reviews and meta‐analyses in 2019 (*n* = 100) stating their source of funding, compared to 85% (*n* = 19) of systematic reviews and meta‐analyses in 2010. There is room for improvements in reporting quality, especially in the eligibility criteria used, risk of bias of individual studies, risk of bias across studies, and additional analyses conducted (all of which were reported by less than 30% of included studies in 2019, and even fewer in 2010; Table 1).

There was slight improvement in the overall reporting of abstracts in 2019 compared to 2010, primarily due to improvements in reporting of review objectives and information sources. No systematic review or meta‐analysis in either year reported their funding sources in the abstract, and only 4% (*n* = 5/127) in 2019 and none in 2010 reported protocol registration information in the abstract. This may indicate limited awareness of PRISMA‐A [18]. Like another study of abstracts in nursing journals, we did not observe noteworthy improvement in abstract reporting between the years [41]. The items that were better reported in our study were similar to those in the abstracts of systematic reviews in dentistry [42]. A study of systematic reviews on psoriasis interventions by Gómez‐García et al. even found a positive correlation between PRISMA‐A adherence and AMSTAR and ROBIS evaluations based on which they proposed a method to predict the methodological quality of systematic reviews using PRISMA‐A scores [43].

### 4.4 Limitations

There are some limitations to this study. While ROBIS and AMSTAR‐2 assessments were completed in duplicate, data collection with PRISMA, with PRISMA‐A, and for study, author, and journal characteristics were performed by a single author. Additionally, we utilized the PRISMA tool published in 2009, as opposed to the more recent PRISMA 2020, which would have allowed for assessment of reporting quality of studies based on more updated evidence.

Another limitation of this study is the inclusion of systematic reviews from dermatology journals only, which would have excluded dermatological systematic reviews of importance from journals of nondermatology focus.

### 4.5 Future direction

The design, conduct, and reporting of systematic reviews in dermatology can be improved. Journals can support reporting quality by requiring and auditing adherence to reporting guidelines. Audits could, for example, be automated with tools similar to StatReviewer [18], although PRISMA is not yet available [44]. Some have suggested that authors self‐assess risk of bias and methodological quality [34]. This process could be augmented by machine‐learning tools similar to Robot‐Reviewer for randomized controlled trials [45, 46].

A novel approach to updating SRs, in the form of living SRs, has been suggested by the Cochrane Collaboration [47]. Uptake of this method might provide an avenue for quality checks to take place, and simultaneously streamline SR production for authors. Furthermore, newer methodologies continue to emerge to trial quicker and simpler ways to synthesize primary data, such as rapid reviews [48, 49], but these review methods serve different use cases and their place in the evidence hierarchy is yet to be solidified.

## 5 CONCLUSION

Most systematic reviews and meta‐analyses published in the 10 highest‐ranking dermatology journals are at high risk of bias and critically low methodological quality in both years, and despite better reporting quality in 2019 compared to 2010, there remains substantial room for improvement.

## AUTHOR CONTRIBUTIONS

**Annapoorani Muthiah**: Data curation; project administration; writing—original draft; writing—review and editing. **Loch Kith Lee**: Data curation; formal analysis; writing—review and editing. **John Koh**: Data curation; writing—review and editing. **Ashly Liu**: Data curation; writing—review and editing. **Aidan Tan**: Conceptualization; project administration; supervision; writing—review and editing.

## CONFLICT OF INTEREST STATEMENT

The authors declare no conflict of interest.

## Supporting information

Supporting information.

Supporting information.

## Data Availability

The data that supports the findings of this study are available in the Supporting Information S1: Material of this article.
