# Supplementary material for: Quality of systematic reviews and meta‐analyses in dermatology
Source: Cochrane Evid Synth Methods. 2024 May 2;2(5):e12056. doi: 10.1002/cesm.12056 (PMC11795973; doi:10.1002/cesm.12056)
Supplement: Supplementary file 1 — Supporting information. [file CESM-2-e12056-s002.docx]

**SUPPLEMENTARY MATERIAL**

*Figure legends*

Figure S1. PRISMA 2020 flow diagram of the study selection process for systematic reviews and meta-analyses published in 2010.

Figure S2. PRISMA 2020 flow diagram of the study selection process for systematic reviews and meta-analyses published in 2019.

*Tables*

**Table 1. Article characteristics of systematic reviews and meta-analyses in peer-reviewed dermatology journals in 2010 (N=27) and 2019 (N=127)**

| **Article characteristic** | **2010** (n (%)) | **2019** (n (%)) | **χ^2^ test for independent proportion / Fisher’s exact test*** | **Difference in proportions**  **%** (95% CI) |
| --- | --- | --- | --- | --- |
| **Dermatological sub-specialty** | | |  |  |
| Cosmetic dermatology | 0 | 6 (4.7) |  |  |
| Medical dermatology | 20 (74.0) | 112 (88.2) |  |  |
| Mohs surgery | 0 | 2 (1.6) |  |  |
| Paediatric dermatology | 1 (3.7) | 4 (3.1) |  |  |
| Surgical dermatology | 0 | 3 (2.4) |  |  |
| Dermatopathology | 6 (22.2) | 0 |  |  |
| **Dermatological sub-specialty** | | | | |
| Acne vulgaris | 1 (3.7) | 3 (2.4) |  |  |
| Alopecia | 1 (3.7) | 10 (7.9) |  |  |
| Atopic dermatitis | 3 (11.1) | 14 (11.0) |  |  |
| Actinic keratosis | 0 | 5 (3.9) |  |  |
| Bullous pemphigoid | 0 | 1 (0.8) |  |  |
| Cellulitis | 0 | 2 (1.6) |  |  |
| Chronic idiopathic urticaria | 0 | 1 (0.8) |  |  |
| Cobb syndrome | 1 (3.7) | 0 |  |  |
| Cosmetic | 0 | 3 (2.4) |  |  |
| Cutaneous leishmaniasis | 1 (3.7) | 0 |  |  |
| Cutaneous lymphomas | 0 | 1 (0.8) |  |  |
| Erythema multiforme | 0 | 2 (1.6) |  |  |
| Extramammary Paget’s disease | 0 | 1 (0.8) |  |  |
| Favre-Racouchot disease | 0 | 1 (0.8) |  |  |
| General dermatology | 3 (11.1) | 13 (10.2) |  |  |
| Hidradenitis suppurativa | 0 | 4 (3.1) |  |  |
| IgA pemphigus | 0 | 1 (0.8) |  |  |
| Infantile haemangiomas | 0 | 1 (0.8) |  |  |
| Keloids | 0 | 1 (0.8) |  |  |
| Leprosy | 0 | 1 (0.8) |  |  |
| Lichen planus | 0 | 2 (1.6) |  |  |
| Mycosis fungoides | 1 (3.7) | 1 (0.8) |  |  |
| Nutritional deficiency | 0 | 1 (0.8) |  |  |
| Papuloerythroderma | 1 (3.7) | 0 |  |  |
| Paraneoplastic pemphigus | 0 | 1 (0.8) |  |  |
| Photodermatoses | 0 | 1 (0.8) |  |  |
| Pityriasis lichenoides | 0 | 1 (0.8) |  |  |
| Pityriasis versicolor | 1 (3.7) | 0 |  |  |
| Prurigo nodularis | 0 | 1 (0.8) |  |  |
| Pruritis | 0 | 1 (0.8) |  |  |
| Psoriasis | 8 (29.6) | 26 (20.5) |  |  |
| Remnant omphalomesenteric duct | 0 | 1 (0.8) |  |  |
| Rosacea | 0 | 1 (0.8) |  |  |
| Scabies | 0 | 1 (0.8) |  |  |
| Seborrheic keratosis | 0 | 1 (0.8) |  |  |
| Skin cancer | 0 | 20 (15.7) |  |  |
| Skin ulcer | 0 | 1 (0.8) |  |  |
| Solar lentigo | 0 | 1 (0.8) |  |  |
| Steven-Johnson syndrome | 0 | 1 (0.8) |  |  |
| Superficial leiomyosarcoma | 0 | 1 (0.8) |  |  |
| Systemic sclerosis | 0 | 1 (0.8) |  |  |
| Ulcus vulvae acutum Lipschutz | 0 | 1 (0.8) |  |  |
| Vitiligo | 0 | 4 (3.1) |  |  |
| Warts | 0 | 1 (0.8) |  |  |
| **Country of first author** | | |  |  |
| Argentina | 1 (3.7) | 0 |  |  |
| Australia | 0 | 5 (3.9) |  |  |
| Austria | 0 | 2 (1.6) |  |  |
| Belgium | 1 (3.7) | 1 (0.8) |  |  |
| Brazil | 0 | 2 (1.6) |  |  |
| Canada | 0 | 4 (3.1) |  |  |
| Chile | 0 | 2 (1.6) |  |  |
| China | 3 (11.1) | 8 (6.3) |  |  |
| Colombia | 0 | 1 (0.8) |  |  |
| Czech Republic | 1 (3.7) |  |  |  |
| Denmark | 0 | 2 (1.6) |  |  |
| Egypt | 0 | 1 (0.8) |  |  |
| France | 5 (18.5) | 4 (3.1) |  |  |
| Germany | 2 (7.4) | 14 (11.0) |  |  |
| Israel and occupied territories | 0 | 4 (3.1) |  |  |
| Italy | 0 | 3 (2.4) |  |  |
| Korea, Republic of (South Korea) | 1 (3.7) | 4 (3.1) |  |  |
| Lebanon | 0 | 1 (0.8) |  |  |
| Nepal | 0 | 1 (0.8) |  |  |
| Netherlands | 3 (11.1) | 5 (3.9) |  |  |
| Norway | 0 | 2 (1.6) |  |  |
| Poland | 0 | 1 (0.8) |  |  |
| Romania | 0 | 1 (0.8) |  |  |
| Singapore | 0 | 1 (0.8) |  |  |
| South Africa | 0 | 2 (1.6) |  |  |
| Spain | 2 (7.4) | 2 (1.6) |  |  |
| Taiwan | 1 (3.7) | 5 (3.9) |  |  |
| Thailand | 0 | 1 (0.8) |  |  |
| United Kingdom | 1 (3.7) | 10 (7.9) |  |  |
| United States of America (USA) | 6 (22.2) | 38 (29.9) |  |  |
| **Number of authors** | | | | |
| Median, IQR | 5.00, 3.00 | 5.00, 3.00 |  |  |
| **Title self-identification** | | |  |  |
| None | 6 (22.2) | 12 (9.4) |  |  |
| Meta-analysis | 4 (14.8) | 7 (5.5) |  |  |
| Systematic review | 15 (55.5) | 54 (42.5) |  |  |
| Systematic review and meta-analysis | 2 (7.4) | 54 (42.5) |  |  |
| **Abstract self-identification** | | |  |  |
| None | 7 (25.9) | 21 (16.5) |  |  |
| Meta analysis | 5 (18.5) | 14 (11.0) |  |  |
| Systematic review | 12 (44.4) | 52 (40.9) |  |  |
| Systematic review and meta-analysis | 3 (11.1) | 40 (31.5) |  |  |
| **PROSPERO status** | | |  |  |
| Prospectively registered in PROSPERO* | 0 | 3 (2.4) |  |  |
| Registered in another database | 0 | 3 (2.4) |  |  |
| Retrospectively registered in PROSPERO* | 0 | 32 (25.2) |  |  |
| Unregistered | 27 (100.0) | 89 (70.1) |  |  |
| **Systematic review individual study type** | | |  |  |
| RCTs and non-RCTs | 10 (37.0) | 47 (37.0) |  |  |
| RCTs only | 6 (22.2) | 23 (18.1) |  |  |
| Non-RCTs only | 11 (40.7) | 57 (44.9) |  |  |
| **Intervention AMSTAR 2** (Systematic reviews of interventions) | | | | |
| Yes | 8 (29.6) | 56 (44.1) | χ^2^(1)=1.92, p=0.16 | 8.61 (-3.0 – 20.3) |
| No | 19 (70.3) | 71 (55.9) |  |  |
| **Search strategy** | | |  |  |
| Yes | 10 (37.0) | 81 (63.8) | χ^2^(1)=6.59, p=0.01 | 16.00 (3.2 – 28.7) |
| No | 17 (62.9) | 46 (36.2) |  |  |
| **Database number** | | |  |  |
| Median, IQR | 3.00, 2.50 | 4.00, 4.00 |  |  |
| **Sample size** | | |  |  |
| Median, IQR | 20.0, 36.5 | 24.0, 37.5 |  |  |
| **Databases searched** | | |  |  |
| Medline/Pubmed | 22 (81.4) | 126 (99.2) |  |  |
| Embase | 13 (48.1) | 87 (68.5) |  |  |
| Cochrane Library | 14 (51.8) | 69 (54.3) |  |  |
| Web of Science | 2 (7.4) | 35 (27.6) |  |  |
| CINAHL | 2 (7.4) | 11 (8.7) |  |  |
| LILACS | 3 (11.1) | 13 (10.2) |  |  |
| SCOPUS | 2 (7.4) | 19 (15.0) |  |  |
| ERIC | 2 (7.4) | 0 |  |  |
| SAGE | 0 | 0 |  |  |
| PsycARTICLES | 0 | 0 |  |  |
| PsycINFO | 0 | 13 (10.2) |  |  |
| Other | 12 (44.4) | 49 (38.5) |  |  |
| **PROSPERO inclusion**^†^ | | |  |  |
| Yes | 0 | 32 (25.2) | χ^2^(1)=8.59, p=0.00 | 22.1 (14.8 – 29.5) |
| No | 27 (100.0) | 95 (74.8) |  |  |
| **Exclusion of non-English text** | | |  |  |
| English and other | 19 (70.3) | 54 (42.5) | χ^2^(1)=6.93, p=0.00 | 16.2 (4.17 – 28.1) |
| English only | 8 (28.6) | 73 (57.5) |  |  |
| **Primary funding source** | | |  |  |
| Self-funded/unfunded | 23 (85.1) | 86 (67.7) |  |  |
| Commercial sector/industry | 2 (7.4) | 11 (8.7) |  |  |
| Charities/societies/foundations | 1 (3.7) | 7 (5.5) |  |  |
| University | 0 | 2 (1.6) |  |  |
| Other collaborative groups | 0 | 2 (1.6) |  |  |
| Government body | 1 (3.7) | 19 (15.0) |  |  |
| **Conflicts of interests** | | |  |  |
| Yes | 8 (29.6) | 39 (30.7) |  |  |
| No | 16 (59.2) | 86 (67.7) |  |  |
| Not declared | 3 (11.1) | 2 (1.6) |  |  |
| **Exclusion of years** | | |  |  |
| No | 16 (59.2) | 97 (76.4) |  |  |
| Yes, with explanation | 2 (7.4) | 13 (10.2) |  |  |
| Yes, without explanation | 9 (33.3) | 17 (13.4) |  |  |
| **Risk of bias of included individual studies** | | |  |  |
| Yes | 8 (29.6) | 76 (59.8) | χ^2^(1)=8.20, p=0.00 | 17.6 (5.4 – 29.8) |
| No | 19 (70.3) | 51 (40.1) |  |  |
| **Risk of bias across included studies** | | |  |  |
| Yes | 5 (18.5) | 31 (24.4) | χ^2^(1)=0.43, p=0.51 | 4.76 (-8.5 - 18.1) |
| No | 22 (81.4) | 96 (75.6) |  |  |
| **Risk of bias tool used** | | |  |  |
| Cochrane Risk of Bias 2.0 tool | 0 | 0 |  |  |
| Cochrane Risk of Bias tool 2008 | 1 (3.7) | 29 (22.8) |  |  |
| SIGN Checklist for RCT | 1 (3.7) | 1 (0.8) |  |  |
| ROBINS-1 | 0 | 2 (1.6) |  |  |
| Newcastle Ottawa Scale | 1 (3.7) | 31 (24.4) |  |  |
| SIGN Checklist for case-control and cohort | 0 | 1 (0.8) |  |  |
| Other | 6 (22.2) | 20 (15.7) |  |  |
| **Meta-analysis**^‡^ | | |  |  |
| Principle summary measures stated | 14 (93.3) | 70 (94.5) |  |  |
| Method synthesis of data MA described | 13 (86.6) | 61 (87.1) |  |  |
| Results of individual studies presented | 9 (60.0) | 63 (86.3) |  |  |
| Present synthesis of results (results of MA) | 13 (86.6) | 61 (87.1) |  |  |
| **Search strategy number of independent reviewers** | | |  |  |
| None | 0 | 30 (23.6) |  |  |
| 1 | 0 | 5 (3.9) |  |  |
| 2 | 16 (59.2) | 83 (65.4) |  |  |
| 3 | 1 (3.7) | 3 (2.4) |  |  |
| Unspecified | 10 (37.0) | 6 (4.7) |  |  |
| **Methodological quality number of independent reviewers** | | | | |
| None | 14 (51.8) | 51 (40.2) |  |  |
| 1 | 0 | 0 |  |  |
| 2 | 10 (37.0) | 38 (29.9) |  |  |
| 3 | 2 (7.4) | 1 (0.8) |  |  |
| Unspecified | 1 (3.7) | 37 (29.1) |  |  |
| **Eligibility criteria** | | |  |  |
| Yes | 18 (66.6) | 116 (91.3) | Fisher’s exact test=0.002 | 31.6 (9.01 – 54.1) |
| No | 9 (33.3) | 11 (8.7) |  |  |
| **Data collection process** | | |  |  |
| Yes | 19 (70.3) | 87 (68.5) | χ^2^(1)=0.03, p=0.84 | -1.2 (-14.1 – 11.6) |
| No | 8 (29.6) | 40 (31.5) |  |  |
| **Data collection items listed** | | |  |  |
| Yes | 18 (66.6) | 87 (68.5) | χ^2^(1)=0.03, p=0.85 | 1.2 (-11.8 – 14.2) |
| No | 9 (33.3) | 40 (31.5) |  |  |
| **Study characteristics** | | |  |  |
| Yes | 17 (62.9) | 93 (73.2) | χ^2^(1)=1.15, p=0.28 | 7.2 (-6.8 – 21.4) |
| No | 10 (37.0) | 34 (26.8) |  |  |
| **Contact with authors** | | |  |  |
| Yes | 4 (14.8) | 34 (26.8) | χ^2^(1)=1.71, p=0.19 | 9.3 (-2.8 – 21.5) |
| No | 23 (85.1) | 93 (73.2) |  |  |
| **Limitations of systematic review** | | |  |  |
| Yes | 19 (70.3) | 111 (87.4) | Fisher’s exact test=0.039 | 18.7 (-1.1 – 38.5) |
| No | 8 (29.6) | 16 (12.6) |  |  |
| **Conclusion summary** | | |  |  |
| Yes | 27 (100.0) | 118 (92.9) | Fisher’s exact test=0.361 | -18.6 (-25.0 – -12.3) |
| No | 0 | 9 (7.1) |  |  |
| **Number of references** | | |  |  |
| Median, IQR | 41.0, 32.0 | 51.0, 28.0 |  |  |
| *Fisher’s exact test was used if any of the expected frequencies were less than 5  ^†^ PROSPERO was launched in February 2011  ^‡^ Sample size of systematic reviews vary in the following rows due to exclusion of systematic reviews which do not include a meta-analyses in their study. | | | | |

| **Table S2. Journal characteristics of peer-reviewed dermatology journals (N=10) containing systematic reviews and meta-analyses in 2010 and 2019.** | |
| --- | --- |
| **Journal Characteristic** | **N(%)** |
| **Year of First Publication** |  |
| 1870 | 1 (10) |
| 1893 | 1 (10) |
| 1938 | 1 (10) |
| 1976 | 1 (10) |
| 1983 | 1 (10) |
| 1992 | 2 (20) |
| 1995 | 1 (10) |
| 2000 | 1 (10) |
| 1888 | 1 (10) |
| **Journal Publisher** |  |
| Adis International Ltd. | 1 (10) |
| American Medical Association | 1 (10) |
| Elsevier Inc. | 3 (30) |
| Lippincott Williams & Wilkins | 1 (10) |
| S. Karger AG | 1 (10) |
| Wiley-Blackwell Publishing Ltd. | 3 (30) |
| **Language of Journal** |  |
| English | 10 (100) |
| **Total Number of Systematic Reviews** |  |
| Mean, IQR | 14.8, 20.0 |
| **Country of Journal** |  |
| Netherlands | 1 (10) |
| Switzerland | 1 (10) |
| United Kingdom | 4 (40) |
| United States of America | 4 (40) |
| **Peer Review Process** |  |
| Double blinded | 1 (10) |
| Single blinded | 7 (70) |
| Unspecified | 2 (20) |
| **PRISMA statement** |  |
| Required | 2 (20) |
| Recommended | 5 (50) |
| No | 3 (30) |
| **Open Access** |  |
| Open Access | 1 (10) |
| Hybrid Open Access | 9 (90) |
| **PROSPERO Registration** |  |
| Explicitly Recommended | 2 (20) |
| Implicitly Recommended | 5 (50) |
| No | 3 (30) |
| **SJR** (mean, IQR) |  |
| SJR 2010 | 1.39, 0.42 |
| SJR 2018 | 1.65, 0.60 |
| **Impact Factor** (mean, IQR) |  |
| Impact factor 2010 | 3.81, 1.24 |
| Impact factor 2019 | 5.33, 3.66 |
| **Number of Publications** (mean, IQR) |  |
| Number of publications 2010 | 315, 270 |
| Number of publications 2019 | 802, 1125 |

n = count; % = percentage; SJR = scientific journal ranking
